# Pepper Rootstock and Scion Physiological Responses Under Drought Stress

**DOI:** 10.3389/fpls.2019.00038

**Published:** 2019-01-28

**Authors:** Lidia López-Serrano, Guillermo Canet-Sanchis, Gabriela Vuletin Selak, Consuelo Penella, Alberto San Bautista, Salvador López-Galarza, Ángeles Calatayud

**Affiliations:** ^1^Departamento de Horticultura, Instituto Valenciano de Investigaciones Agrarias, Valencia, Spain; ^2^Department of Plant Science, Institute for Adriatic Crops and Karst Reclamation, Split, Croatia; ^3^Departamento de Producción Vegetal, Universitat Politècnica de València, Valencia, Spain

**Keywords:** drought, gas exchange, grafted, oxidative stress, pepper, rootstock, water relations

## Abstract

In vegetables, tolerance to drought can be improved by grafting commercial varieties onto drought tolerant rootstocks. Grafting has emerged as a tool that copes with drought stress. In previous results, the A25 pepper rootstock accession showed good tolerance to drought in fruit production terms compared with non-grafted plants and other rootstocks. The aim of this work was to study if short-term exposure to drought in grafted plants using A25 as a rootstock would show tolerance to drought now. To fulfill this objective, some physiological processes involved in roots (rootstock) and leaves (scion) of grafted pepper plants were analyzed. Pepper plants not grafted (A), self-grafted (A/A), and grafted onto a tolerant pepper rootstock A25 (A/A25) were grown under severe water stress induced by PEG addition (-0.55 MPa) or under control conditions for 7 days in hydroponic pure solution. According to our results, water stress severity was alleviated by using the A25 rootstock in grafted plants (A/A25), which indicated that mechanisms stimulated by roots are essential to withstand stress. A/A25 had a bigger root biomass compared with plants A and A/A that resulted in better water absorption, water retention capacity and a sustained CO_2_ assimilation rate. Consequently, plants A/A25 had a better carbon balance, supported by greater nitrate reductase activity located mainly in leaves. In the non-grafted and self-grafted plants, the photosynthesis rate lowered due to stomatal closure, which limited transpiration. Consequently, part of NO_3_^-^ uptake was reduced in roots. This condition limited water uptake and CO_2_ fixation in plants A and A/A under drought stress, and accelerated oxidative damage by producing reactive oxygen species (ROS) and H_2_O_2_, which were highest in their leaves, indicating great sensitivity to drought stress and induced membrane lipid peroxidation. However, drought deleterious effects were slightly marked in plants A compared to A/A. To conclude, the A25 rootstock protects the scion against oxidative stress, which is provoked by drought, and shows better C and N balances that enabled the biomass to be maintained under water stress for short-term exposure, with higher yields in the field.

## Introduction

In agriculture, drought stress is one of the most limiting factors for growing crops, mainly due to a poor plant carbon balance, which is largely dependent on photosynthesis (Flexas et al., 2009). This is associated with a significant drop in the leaf water potential and transpiration (Fahad et al., 2017) which, in turn, affect nutrient absorption. These restrictions make plants more susceptible to photo damage by increasing reactive oxygen species (ROS) that may damage the cellular membrane and other vital molecules like DNA, lipids, and proteins (Fahad et al., 2017). Metabolic alterations in plants by drought lead to significant yield reductions, which imply major economic loss and affect global food security. Bearing in mind that most climate change scenarios predict a more drought incidences, it is necessary to increase food production to satisfy the population’s demand (Fahad et al., 2017).

The selection of tolerant genotypes is a considerable challenge to improve productivity with limited water resources. Conventional plant breeding has had limited success at mitigating the effects of abiotic stress on plant productivity (Gilliham et al., 2017; Lamaoui et al., 2018). This can be ascribed to both the complexity of traits and lack of appropriate selection tools (Ashraf and Foolad, 2007; Schwarz et al., 2010). In addition, it is very difficult to combine enhanced yields and superior product quality with tolerance to drought and other abiotic stresses (Finckh, 2008; Lammerts van Bueren et al., 2011).

Genetic transformation could prove a powerful tool in plant breeding (Borsani et al., 2003; Cuartero et al., 2006; Martínez-Rodríguez et al., 2008). However, lack of public acceptance of genetic engineering clearly indicates the need for alternative strategies to enhance abiotic stress tolerance (Munns, 2002; Estañ et al., 2005).

One possible solution to cope with abiotic stress and reduce production losses involves using graft technology (Rivero et al., 2003a; Colla et al., 2010; Savvas et al., 2010; Schwarz et al., 2010; Sánchez-Rodríguez et al., 2014). Some studies have demonstrated the efficiency of tolerant rootstocks in reducing the effects of drought on the scion by improving physiological performance and productivity through different approaches, like using a larger and vigorous root rootstock system capable of absorbing water and nutrients, and maintaining the root relative growth rate and leaf-relative water content more efficiently than non-grafted plants. This behavior has been observed in tomato (Sánchez-Rodríguez et al., 2012; Yao et al., 2016) and watermelon (Chouka and Jebari, 1999; Alan et al., 2007; Rouphael et al., 2008). Another alternative is active osmotic adjustment as it can contribute to improve the uptake of more water mediated by the accumulation of a range of osmotically active molecules, as reported in pepper (Anjum et al., 2012; Penella et al., 2014b) and tomato (Yao et al., 2016) grafted plants. In addition, plants subjected to drought stress tend to overproduce ROS; the activation and/or modulation of an antioxidant defense system plays an important role in conferring tolerance under drought and constitutes the first line of defense by reducing damage by lipid peroxidation in tomato grafted plants (Yao et al., 2016). The ability to limit water loss can help to maintain the photosynthesis rate and improve NO_3_^-^ assimilation in grafted plants under water deficit by allowing both plant growth and productivity (Rouphael et al., 2008; Sánchez-Rodríguez et al., 2013; Penella et al., 2014b).

Recently, the number of reports on grafting as a mean to improve tolerance to drought has increased in mainly tomato, watermelon and cucumber (Kumar et al., 2017). Nevertheless, the use of rootstocks that tolerate abiotic stresses is lacking in pepper plants because available commercial rootstocks provide limited profits (Lee et al., 2010; Penella et al., 2014a; Kyriacou et al., 2017). Overall screenings to detect tolerant Capsicum plants are necessary to use them as rootstocks (Penella et al., 2014a).

In our previous experiments, after wide screening of Capsicum accessions, a drought-tolerant genotype was selected to be used as a rootstock. It was tested in productivity terms (A25 code) and showed an increase in marketable fruit of 118% *versus* non-grafted plants (Penella et al., 2017).

Thus present work aimed to (i) determine if short-term drought stress exposure of seedling grafted plants onto A25 could express its tolerance to determine the highest productivity (Penella et al., 2017); (ii) identify rootstock and scion physiological traits associated with drought tolerance to open up new strategies that improve crop performance under limited water supply conditions. Very few studies on simultaneous changes in the rootstock/scion are available, but none about the perception of scions and rootstock remodeling can be found (Li et al., 2014; Liu et al., 2016). To fulfill these objectives, we compared the behavior of photosynthetic, water relations, antioxidant mechanisms and oxidative index stress in non-grafted and self-grafted plants, and in those grafted onto a tolerant rootstock (A25), under drought stress and control conditions.

## Materials and Methods

### Experimental Site and Greenhouse Conditions

The experiment was conducted in a Venlo-type glasshouse located in Moncada (Valencia, Spain; Latitude: 39.58951793357715, Longitude: -0.3955507278442383, 37 m above sea level) at the IVIA research institute.

During the experiments, plants were grown under natural light conditions with a maximum PAR of 1000 μmol m^-2^ s^-1^ (800–1,000 μmol m^-2^ s^-1^), a mean temperature of 22°C (18–25°C) and a mean humidity of 60% (50–70%).

### Plant Material and Management

Based on previous studies, a pepper accession of *Capsicum annuum* L. was used as a tolerant rootstock to water scarcity (code A25). Pepper cultivar “Adige” (code A) (Lamuyo type, Sakata Seeds, Japan) was used as a scion. Seeds were sown in March 2016 in 54-hole seed trays filled with enriched substrate for germination. Three months after sowing, plants were grafted by the tube-grafting method (Penella et al., 2014b).

Three weeks after grafting (June 2016), seedlings were removed from substrate, and their roots were cleaned before being placed in 5 L polyethylene pots covered with aluminum sheets. Pots were filled with a nutrient solution containing (in mmol L^-1^): 12.3 NO_3_^-^, 1.02 H_2_PO_4_, 2.45 SO_4_^2-^, 3.24 Cl^-^, 0.6 NH_4_^+^, 5.05 K^+^, 4.23 Ca^2+^, 2.55 Mg^2+^, 2.2 Na^+^ and micronutrients (15.8 μM Fe^2+^, 10.3 μM Mn^2+^, 4.2 μM Zn^2+^, 43.5 μM B^+^, 2.14 μM Cu^2+^), which were artificially aerated with an air pump. Electrical conductivity and pH were 2.14 dS m^-1^ and 6.7, respectively. Nutrient solution was added daily to compensate absorption. The water stress treatment was induced after a 7-day seedling acclimation in pots by adding 5% PEG 8000 (Sigma Co.) to the nutrient solution. The osmotic potential of the solutions, measured by a vapor osmometer (Digital osmometer, Wescor, Logan, UT, United States), was -0.55 MPa for 5% PEG and -0.05 MPa for the control solution (0% PEG).

The assay was based on three plant combinations: A (non-grafted plants of cultivar Adige), A/A (A grafted onto itself by showing the graft effect) and A/A25 (A grafted onto the A25 rootstock). The layout was completely randomized with four replications for each combination and six plants per replication.

All the physiological measurements were taken 1, 2, 4, and 7 days after treatment began (DAT). Measurements were taken in fully and expanded mature leaves (third to fourth leaf from the shoot apex), and also in lateral roots for some physiological measurements. The layout was randomized with 12 measurements (three plants per replication) per plant combination and treatment for the gas exchange measurement, and with four measurements (one plant per replication) in the other analysis of the physiological parameters. Biomass determinations were made only on 7 DAT using the plants that were not involved in the physiological measurements. Eight plants per plant combination and treatment were analyzed (two plants per replication).

### Gas Exchange Measurements

The CO_2_ assimilation rate (*A*_N_, μmol CO_2_ m^-2^ s^-1^), stomatal conductance to water vapor (*g*_s_, mol H_2_O m^-2^ s^-1^), and substomatal CO_2_ concentration (*C*_i_, μmol CO_2_ mol^-1^ air) were measured with a portable LI-COR 6400 infrared gas analyzer (Li-Cor Inc., United States). Measurements were taken under saturating light conditions (1,000 μmol quanta m^-2^ s^-1^), with reference CO_2_ (400 μmol CO_2_ mol^-1^) at 24°C (24°C ± 2) and 75% relative humidity (75% ± 10). The fully expanded (third to fourth leaf from the apex) and non-detached leaves were used for the measurements taken from 09:00 h to 11:00 h (UT + 01:00 h).

### Biomass Determination

Root length and the fresh weight of roots and leaves were measured at the end of the experiment (7 DAT). Fresh roots and leaves were dried at 65°C for 72 h to determine dry weight.

### Water Relations

Relative water content (RWC) was measured by weighing leaves before and after a 24 h rehydration with distilled water. Next they were dried at 65°C for 72 h and the measurement was repeated. RWC was determined by the equation RWC = (FW - DW)/(TW - DW) × 100, where FW, DW, and TW are fresh weight, dry weight, and turgid weight, respectively.

The leaf water potential at pre-dawn (Ψ_w_) was measured with a Schlolander pressure chamber (Wescor Model 600, PMS Instruments, Albany, NY, United States) on detached fresh and mature leaves inside a greenhouse.

The osmotic potential of leaf sap (Ψ_s_ in MPa) was measured by an osmometer (Digital osmometer, Wescor, Logan, UT, United States). Leaves were detached, placed inside 1 mL tubes and quickly frozen at -20°C. After melting, sap was collected by centrifugation at 9,000 rpm for 1 min in 1.5 mL tubes to be used for the osmometer measurements. Osmolyte content (mmol kg^-1^) was converted into MPa by the Van’t Hoff equation.

### Nitrate Reductase Activity

Nitrate reductase activity (Enzyme Code 1.7.1.1) was determined *in vivo* following the methods described by Hageman and Hucklesby (1971) and Jaworski (1971). Disks of 1 cm diameter from mature fresh leaves or 1 cm root pieces were collected. Samples (0.2 g) were suspended in plastic vials containing 10 mL of 100 mM potassium phosphate buffer (pH 7.5), 1% (v/v) n-propanol and 100 mM KNO_3_. Plant samples were incubated in a water bath at 30°C for 60 min in the dark and placed in a boiling water bath for 5 min to stop the enzymatic reaction. The nitrite released from plant material was determined colorimetrically at 540 nm (spectrophotometer PerkinElmer, Lambda 25) by adding 0.02% (w/v) *N*-naphthyl-ethylenediamine and 1% (w/v) sulfanilamide. A standard curve with KNO_2_ was prepared to calculate the amount of NO_2_ contained in the samples.

### Determination of DPPH Radical-Scavenging Capacity

Determination of radical scavenging capacity (RSA) was carried out by the 2,2-diphenyl-1-picrylhydrazyl (DPPH) radical scavenging method, proposed by Brand-Williams et al. (1995) with modifications. First 0.1 g of sample (leaves and roots) was frozen in liquid nitrogen and stored at -80°C. Samples were ground by a mortar with the addition of 80% (v/v) methanol. After 12 h at 4°C in a mixer, samples were centrifuged for 10 min at 10,000 × *g* and 4°C. A 10 μL volume of sample and 990 μL of 0.065 mM DPPH were taken and incubated for 30 min in the darkness at room temperature. Absorbance was measured at 515 nm. The percentage of inhibition of the DPPH radical was measured by the equation: [(DPPH absorption - sample absorption)/DPPH absorption] × 100.

### Total Phenolic Content Analysis

Total phenolic content was determined according to Koç et al. (2010) with modifications. The fresh leaf and root samples (0.1 g) were frozen in liquid nitrogen and stored at -80°C. They were mixed with 1.5 mL of extraction solution [50% (v/v) methanol and 1% (v/v) HCl]. Samples were extracted in a boiling bath at 80°C for 15 min. Then 0.1 mL of root extract and 0.02 mL of leaf extract (diluted in 0.08 mL extraction solution) were mixed with 0.7 mL of Folin–Ciocalteu solution (Sigma-Aldrich^®^), diluted in the 1:10 proportion, and with 0.7 mL of 6% (w/v) Na_2_CO_3_. Samples were incubated at room temperature and in the darkness for 1 h before being subjected to absorbance measurement at 765 nm. Gallic acid was used as a standard.

### Determination of Hydrogen Peroxide

H_2_O_2_ content was determined according to Sergiev et al. (1997) and Velikova et al. (2000) with slight modifications. First 0.25 g of FW (leaves and roots) was frozen in liquid nitrogen and conserved at -80°C. Samples were ground with a mortar and 2 mL of 0.1% (w/v) trichloroacetic acid (TCA). The homogenate was centrifuged at 10,000 × *g* at 4°C for 8 min. With the root samples, 1 mL of the supernatant was added to 0.5 mL of 100 mM potassium phosphate buffer (pH = 7) and 2 mL of 1M KI. For another set of samples, 0.4 mL of leaves was diluted with 0.6 mL of 0.1% (w/v) TCA. Samples were incubated for 1 h at room temperature under dark conditions. Absorbance was measured at 390 nm. H_2_O_2_ content was given by a H_2_O_2_ standard curve.

### Lipid Peroxidation Analysis

Lipid peroxidation was estimated through malondialdehyde (MDA) determinations by the thiobarbituric acid reaction, according to the protocol reported by Heath and Packer (1968), and modified in Dhindsa et al. (1981). First 0.1 g of sample (leaves and roots) was frozen in liquid nitrogen and kept at -80°C. Samples were ground with a mortar and 2 mL of 0.1% (w/v) TCA. Later the homogenate was centrifuged at 10,000 × *g* and 4°C for 5 min. Afterward, 2 mL of reaction buffer (TCA 20% + TBA 0.5%) were added and heated at 95°C for 30 min. The non-specific background absorbance reading at 600 nm was subtracted from the specific absorbance reading at 532 nm.

### Statistical Analysis

The experiment was completely randomized, and every time measurements were separately subjected to a two-way ANOVA (Statgraphics Centurion for Windows, Statistical Graphics Corp.), where plant combinations and treatments were the factors of the analyses. After verifying the significance of the interaction for each variable (data not shown), a one-way ANOVA was performed by joining the plant combination and treatment. Means were compared by the Fisher’s least significance difference (LSD) test at *P* < 0.05. There were no significant differences among replicates for each measured parameter.

## Results

### Gas Exchange Measurements

**FIGURE 1:**
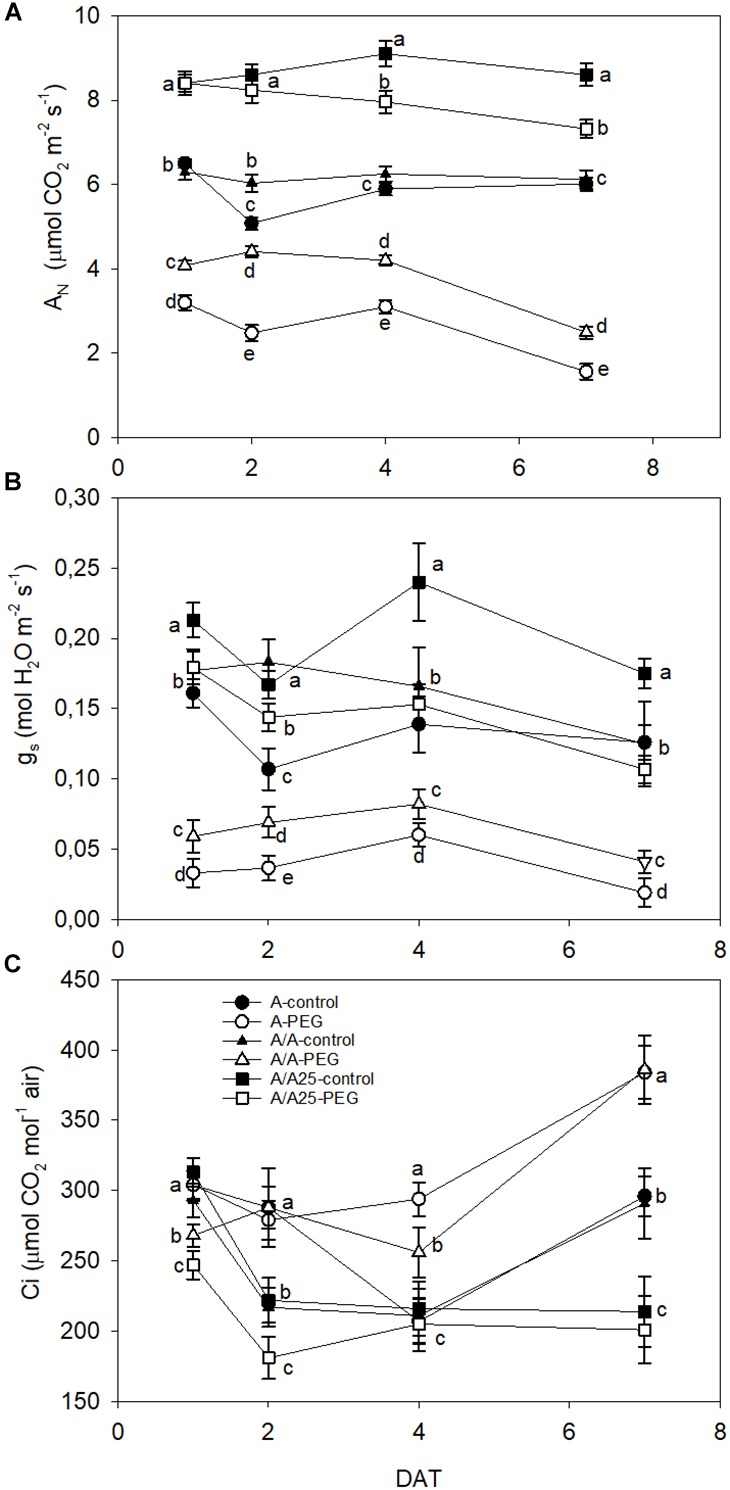
Net CO_2_ assimilation rate (*A*_N_; μmol CO_2_ m^-2^ s^-1^) **(A)**; leaf stomatal conductance (*g*_s_; mol H_2_O m^-2^ s^-1^) **(B)** and substomatal CO_2_ concentration (*C*_i_; μmol CO_2_ mol^-1^ air) **(C)** in the non-grafted pepper plants (cultivar Adige, A), self-grated plants (A/A), and plants grafted onto A25 (A/A25) at 0% PEG (control) or 5% PEG (water stress). Measurements were taken on 1 DAT, 2 DAT, 4 DAT, and 7 DAT (days after treatment with PEG began). Data are the mean values for *n* = 12 ± SE. For each studied time, different letters indicate significant differences at *P* < 0.05 (LSD test).

At 24 h after PEG addition, *A*_N_ dramatically dropped in the A plants, followed by the A/A plants compared to their control (Figure 1A), while plants A/A25 showed no significant differences between PEG treatment and the control. At the end of the experiment (7 DAT), all the plant combinations displayed significant differences with their control. The highest values went to the A/A25 control, followed by A/A25 PEG. The *g*_s_ values (Figure 1B) changed significantly from the beginning of the experiment in all the plant combinations, when low *g*_s_ values were recorded for plants A and A/A under PEG. Parameter *C*_i_ was higher in plants A and A/A under the PEG conditions on 4 DAT and 7 DAT (Figure 1C), but showed no significant differences for plants A/A25.

### Biomass Parameters

**FIGURE 2:**
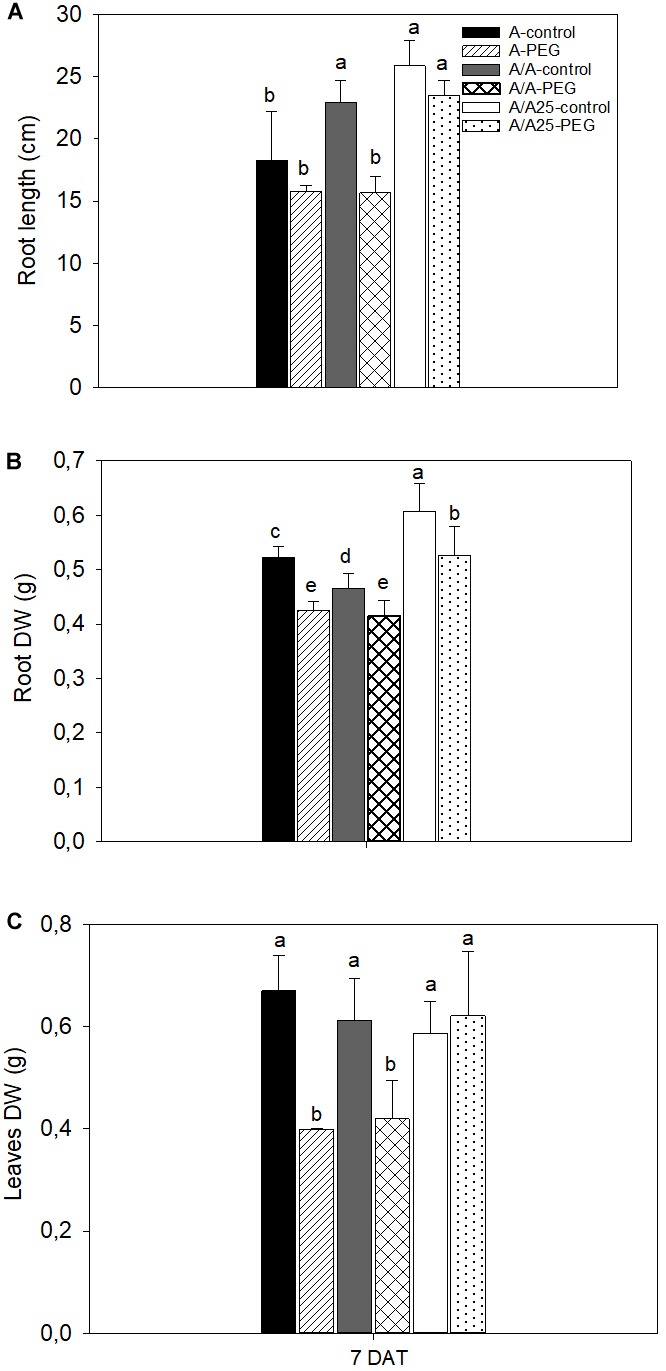
Root length **(A)**, root dry weight (DW) **(B)**, and leaf dry weight **(C)** in the non-grafted pepper plants (cultivar Adige, A), self-grated plants (A/A), and plants grafted onto A25 (A/A25) at 0% PEG (control) or 5% PEG (water stress). Measurements were taken at the end of the experiment, 7 days after treatment with PEG began (7 DAT). Data are the mean values for *n* = 8 ± SE. For each studied time, different letters indicate significant differences at *P* < 0.05 (LSD test).

At the end of the experiment (7 DAT), root length (Figure 2A), root DW (Figure 2B), and leaf DW (Figure 2C) decreased, with significant differences in the A and A/A plants exposed to stress compared with their control treatments. The A/A25 plants exposed to PEG underwent changes in root DW, with a significant decrease compared to the control plants, which was not the case for the other biomass parameters (root length and leaf DW). Under the control conditions, the biomass parameters were higher in the A/A25 plants for the root traits compared with plants A and A/A.

### Hydric and Osmotic Relations

**FIGURE 3:**
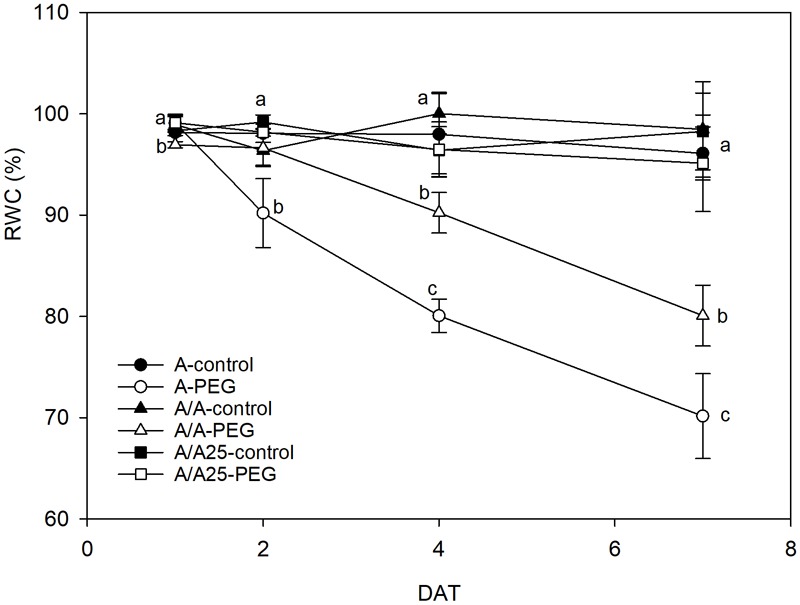
Effect of PEG addition at 0% (control) and 5% (water stress) on relative water content (RWC) on 1 DAT, 2 DAT, 4 DAT, and 7 DAT (days after treatment with PEG began) in the non-grafted pepper plants (cultivar Adige, A), self-grated plants (A/A), and plants grafted onto A25 (A/A25). Data are the mean values for *n* = 4 ± SE. For each studied time, different letters indicate significant differences at *P* < 0.05 (LSD test).

During the experiment, the pepper plants under the control conditions maintained a constant leaf RWC (Figure 3) above a value of 95%. The presence of PEG in the nutrient solution provoked a reduction in RWC from 2 DAT, which became more evident on 7 DAT for the A plants, followed by the A/A plants. The RWC in the A/A25 plants exposed to drought stress remained stable with similar values in the control plants during the experiment.

**FIGURE 4:**
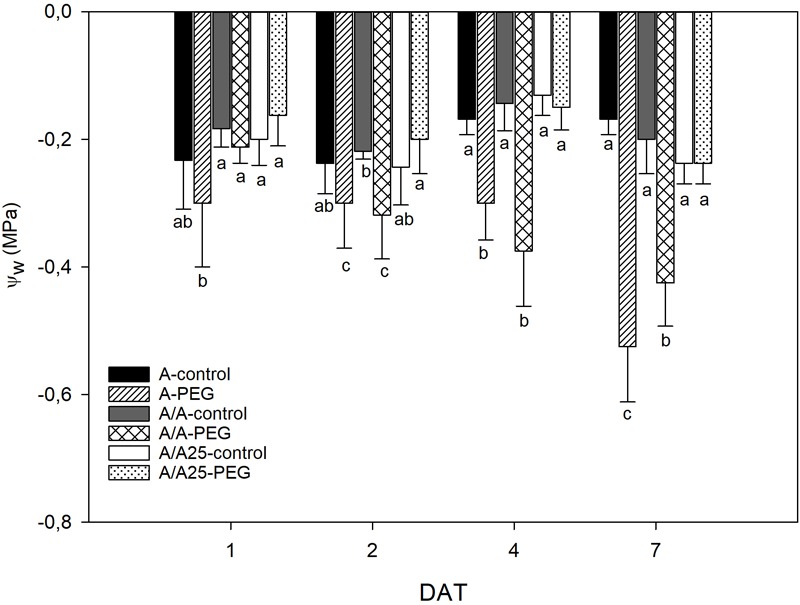
Leaf water potential (MPa) in the non-grafted pepper plants (cultivar Adige, A), self-grated plants (A/A), and plants grafted onto A25 (A/A25) after addition of 0% PEG addition (control) and 5% PEG on 1 DAT, 2 DAT, 4 DAT, and 7 DAT (days after treatment with PEG began). Data are the mean values for *n* = 4 ± SE. For each studied time, different letters indicate significant differences at *P* < 0.05 (LSD test).

The leaf water potential (Ψ_W_) in the control plants did not show any significant difference for each measured time (Figure 4). The A plants under PEG showed changes in Ψ_W_ after 24 h after exposure to stress (1 DAT), which remained stable on 2 DAT and 4 DAT, when both had similar values. Then a sharp drop was observed on 7 DAT. In the A/A plants under the stress conditions, the drop in Ψ_W_ started on 2 DAT and reached a maximum decrease on 7 DAT. For the A/A25 plants under PEG, the Ψ_W_ values during the experiment were similar to the control plants.

**FIGURE 5:**
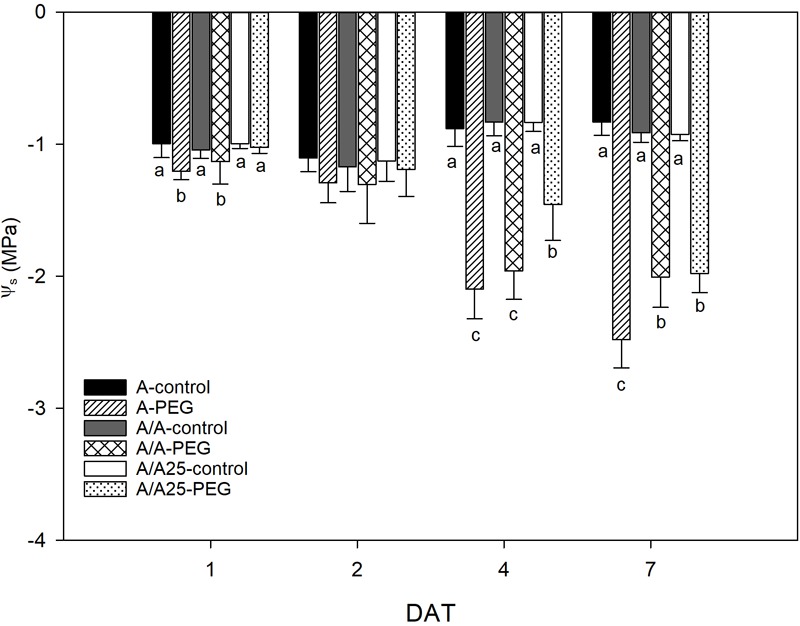
Leaf osmotic potential (MPa) in the non-grafted pepper plants (cultivar Adige, A), self-grated plants (A/A), and plants grafted onto A25 (A/A25) after addition of 0% PEG (control) and 5% PEG on 1 DAT, 2 DAT, 4 DAT, and 7 DAT (days after treatment with PEG began). Data are the mean values for *n* = 4 ± SE. For each studied time, different letters indicate significant differences at *P* < 0.05 (LSD test). The absence of letters on 2 DAT means no significant difference for both factors (plant and treatment).

The Ψ_s_ (Figure 5) lowered in relation to the exposure time to PEG. Within the first measured time frame (1 DAT), plants A and A/A displayed a drop in Ψ_s_ under the stress conditions, which was not found for plants A/A25. On 7 DAT, the maximal decrease was found for A, followed by plants A/A and A/A25.

### Nitrate Reductase Activity

**FIGURE 6:**
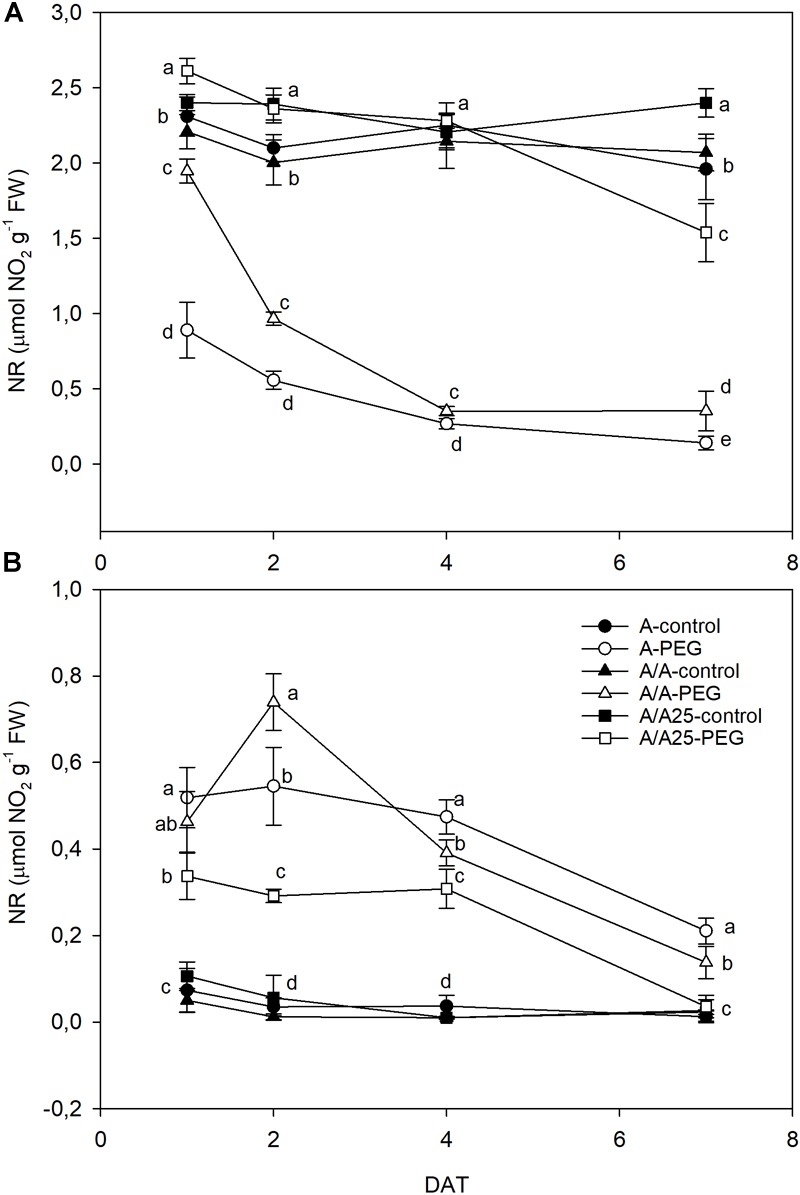
Nitrate reductase activity (NR) in the leaves **(A)** and roots **(B)** of the non-grafted pepper plants (cultivar Adige, A), self-grated plants (A/A), and plants grafted onto A25 (A/A25) under the control conditions (0% PEG) and at 5% PEG on 1 DAT, 2 DAT, 4 DAT, and 7 DAT (days after treatment with PEG began). Dates are the mean values for *n* = 4 ± SE. For each studied time, different letters indicate significant differences at *P* < 0.05 (LSD test).

Nitrate reductase activity (NR) was higher in leaves (Figure 6A) than in roots (Figure 6B). In general terms, the opposite behavior was observed between both organs in activity terms during the experiment, with higher values in leaves that matched the lower values in roots for each time and plant combination. In leaves (Figure 6A), NR dramatically lowered 24 h after adding PEG to the A plants, but this decrease in the A/A plants was less marked compared with the A plants. On 2 DAT, the A/A plants underwent a sharp drop in NR activity under water stress, and the A/A25 plants underwent a reduction only on 7 DAT. In roots (Figure 6B), NR activity increased in all the pepper plant combinations under drought stress compared with the control plants from 1 DAT to 2 DAT. Afterward, activity lowered until 7 DAT, when the lowest values were obtained in the A/A25 plants, followed by A/A and finally A.

### DPPH-Radical Scavenging Activity

**FIGURE 7:**
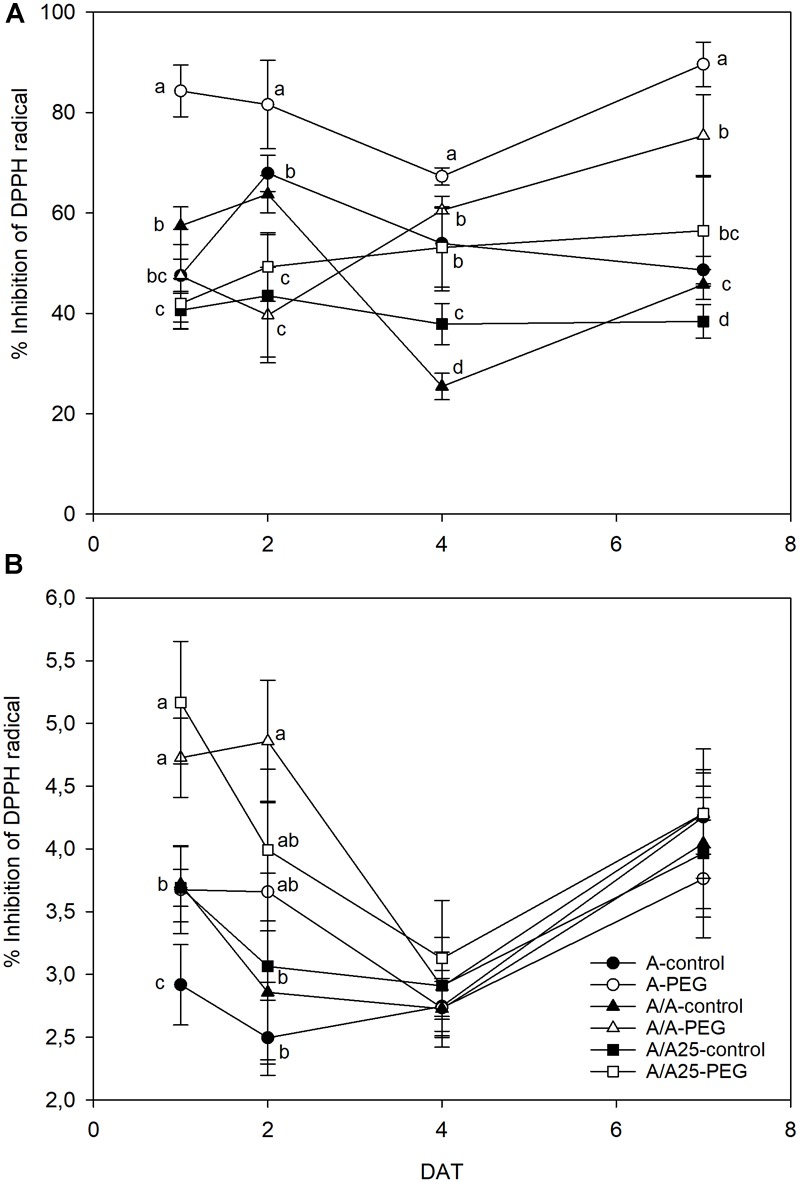
Percentage of inhibition of DPPH radical in the leaves **(A)** and roots **(B)** of the non-grafted pepper plants (cultivar Adige, A), self-grated plants (A/A), and plants grafted onto A25 (A/A25) under the control conditions (0% PEG) and 5% PEG on 1 DAT, 2 DAT, 4 DAT, and 7 DAT (days after treatment with PEG began). Data are the mean values for *n* = 4 ± SE. For each studied time, different letters indicate significant differences at *P* < 0.05 (LSD test). In **(B)**, the absence of letters on 7 DAT means no significant difference for both factors (plant and treatment).

The leaves of the plants grown under drought stress displayed an increasing percentage of inhibition of the DPPH radical (Figure 7A). For the A plants under PEG, the increase was recorded earlier (1 DAT), whereas DPPH-RSA started from 4 DAT in A/A and A/A25. For the plants under drought stress on 7 DAT, maximum activity was found for the A plants followed by A/A, with minor activity for A/A25. DPPH-RSA was higher in leaves than in roots (Figure 7). In roots, major activity was exhibited on 1 DAT and 2 DAT in the plants exposed to PEG (Figure 7B). Afterward no significant differences were found in activity among treatments.

### Total Phenolic Content

**FIGURE 8:**
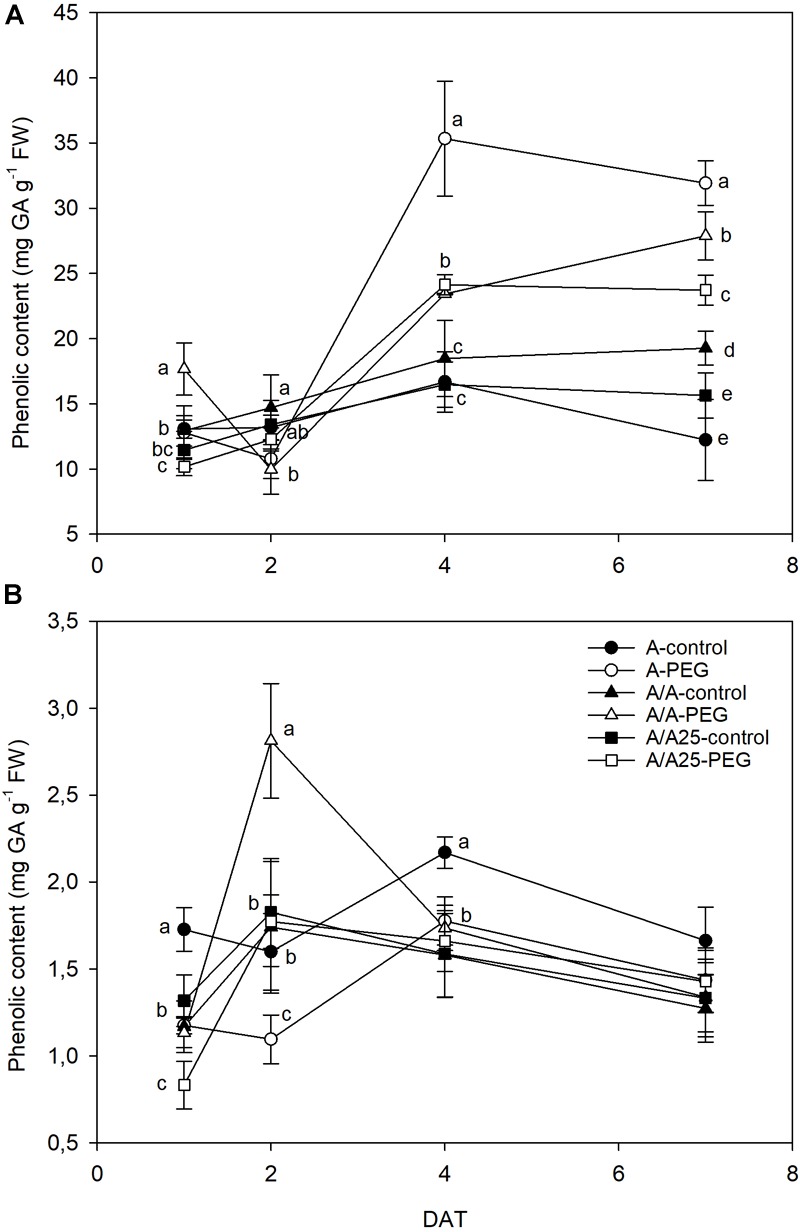
Changes in phenolic content in the leaves **(A)** and roots **(B)** of the non-grafted pepper plants (cultivar Adige, A), self-grated plants (A/A), and plants grafted onto A25 (A/A25) under the control conditions (0% PEG) and 5% PEG on 1 DAT, 2 DAT, 4 DAT, and 7 DAT (days after treatment with PEG began). Data are the mean values for *n* = 4 ± SE. For each studied time, different letters indicate significant differences at *P* < 0.05 (LSD test). In **(B)**, the absence of letters on 7 DAT means no significant difference for both factors (plant and treatment).

An increase in phenolic content was observed during the experiment in the leaves of the plants exposed to drought stress (Figure 8A). The most marked phenolic increase was found for plants A in the PEG treatment, with significant differences shown on 4 DAT and 7 DAT compared to the control plants. In roots (Figure 8B), phenolic contents were 10-fold lower than in leaves, and values were similar among treatments, except for the A/A plants under drought stress at 2 DAT, when a sharp drop was observed.

### H_2_O_2_ Concentration

**FIGURE 9:**
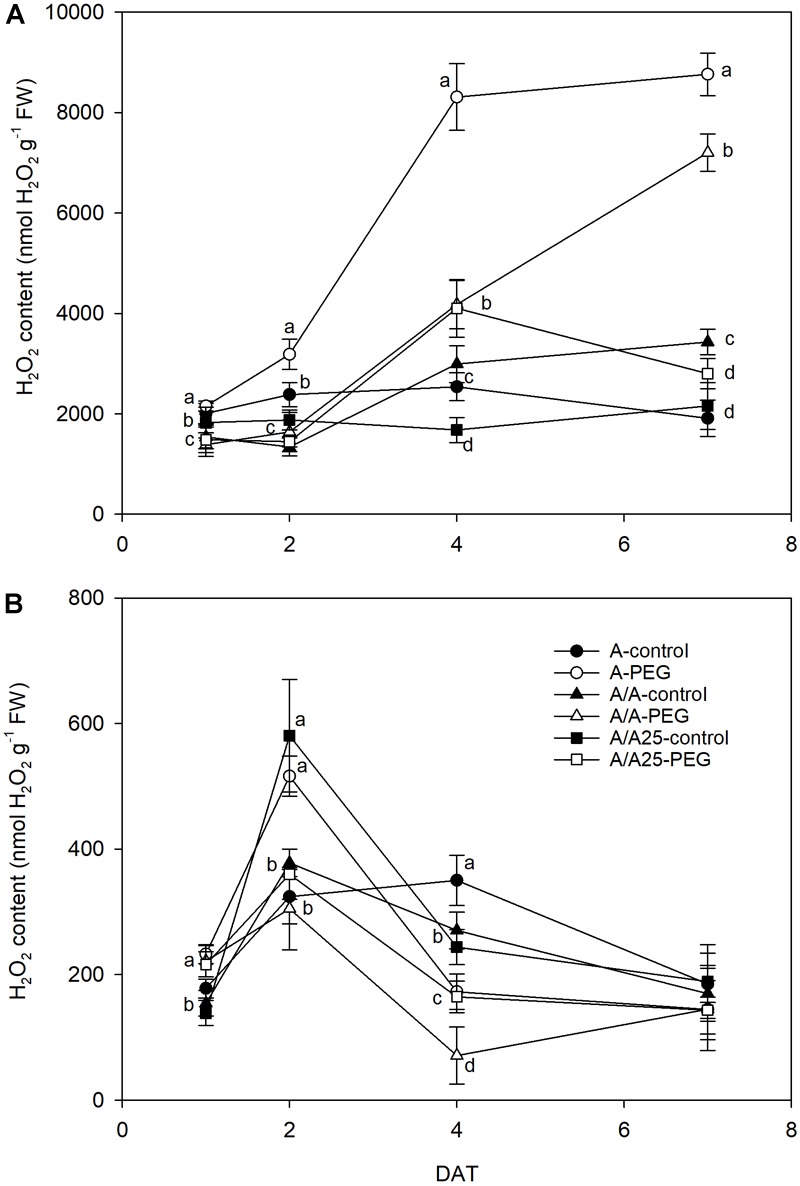
Hydrogen peroxide concentration in the leaves **(A)** and roots **(B)** of the non-grafted pepper plants (cultivar Adige, A), self-grated plants (A/A), and plants grafted onto A25 (A/A25) under the control conditions (0% PEG) and 5% PEG on 1 DAT, 2 DAT, 4 DAT, and 7 DAT (days after treatment with PEG began). Data are the mean values for *n* = 4 ± SE. For each studied time, different letters indicate significant differences at *P* < 0.05 (LSD test). In **(B)**, the absence of letters on 7 DAT means no significant difference for both factors (plant and treatment).

The hydrogen peroxide level in leaves (Figure 9A) increased after exposing plants to drought stress from 4 DAT to 7 DAT, which was emphasized mainly in plants A and A/A with significant differences compared to the control plants. In roots (Figure 9B), the H_2_O_2_ concentration was approximately 10-fold lower than in leaves. The maximum concentrations for all the plant combinations were recorded on 2 DAT. At the end of the experiments, no significant differences were observed between plants and treatments.

### Lipid Peroxidation

**FIGURE 10:**
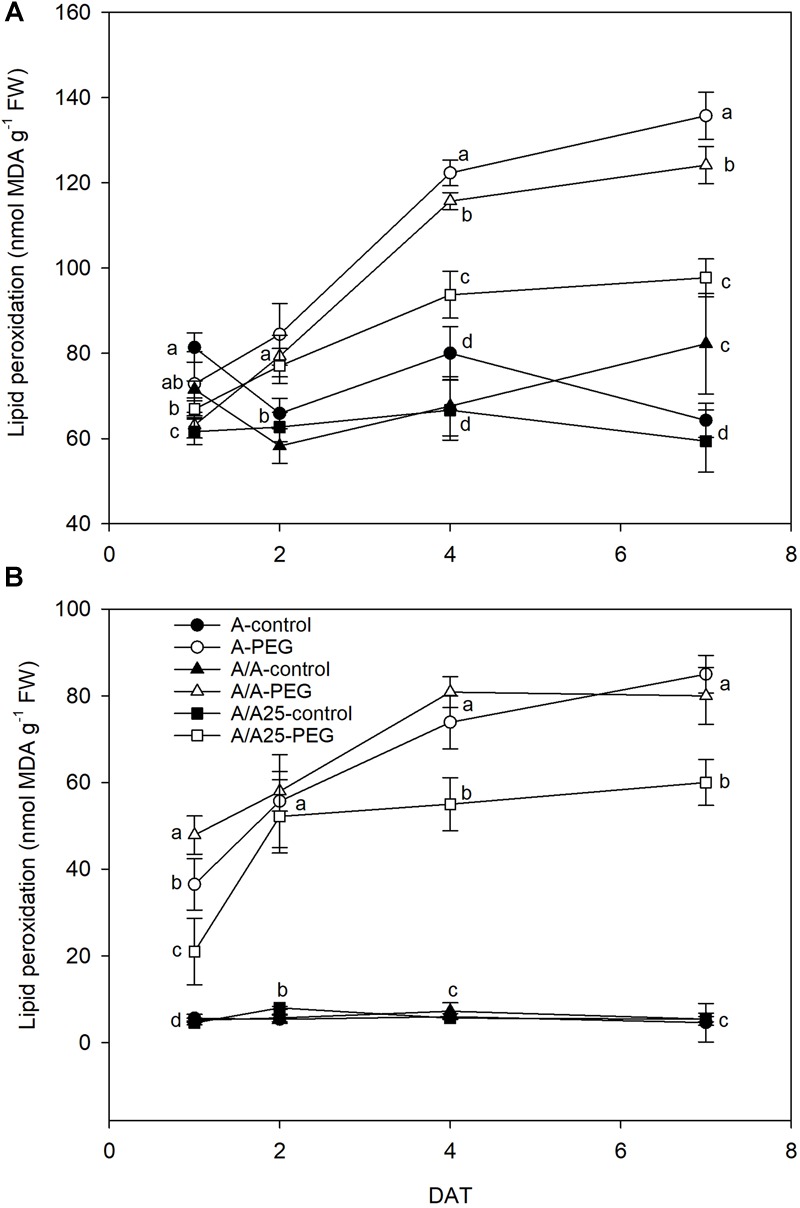
Malondialdehyde content (MDA) of the leaves **(A)** and roots **(B)** of the non-grafted pepper plants (cultivar Adige, A), self-grated plants (A/A), and plants grafted onto A25 (A/A25) under the control conditions (0% PEG) and 5% PEG on 1 DAT, 2 DAT, 4 DAT, and 7 DAT (days after treatment with PEG began). Data are the mean values for *n* = 4 ± SE. For each studied time, different letters indicate significant differences at *P* < 0.05 (LSD test).

The MDA concentration in leaves increased with time from 2 DAT (Figure 10A). As a result, the highest MDA levels were found in the A plants, followed by the A/A plants under drought stress, and the A/A25 plants on 7 DAT. In roots (Figure 10B), lipid peroxidation increased and followed this trend in leaves during the experiment. On 7 DAT, the highest values were recorded for pepper plants A and A/A under drought stress.

## Discussion

In this experiment, we observed numerous changes in the biochemical and physiological processes during the short drought stress treatment, which were provoked by the addition of 5% PEG. These processes were involved in the perception and transduction of stress in the scion depending on the employed rootstock. We found that pepper plants with the A25 rootstock (A/A25) displayed greater tolerance to drought stress in this short-term experiment, as indicated by the effects on the scion in terms of biomass conservation, photosynthesis and RWC maintenance, lower lipid peroxidation and greater NR activity in leaves compared with the non-grafted and self-grafted pepper plants. The fact that the scion (A) suffered less drought stress was related to the better tolerance of root parts (A25), which encompassed the greater vigor root system and the lower H_2_O_2_ concentration, lipid peroxidation and NR activity. These results indicated that scion performance is largely dependent on the tolerance of roots to drought stress, although the root to shoot communication involved in scion pepper stress responses is largely unknown.

In whole-plant terms, the effect of abiotic stresses is usually noticed as a reduction in photosynthesis and growth, and is associated with alterations in carbon and nitrogen metabolism (Loggini et al., 1999; García-Mata and Lamattina, 2001). To support this idea, in our experiment drought stress negatively affected the aboveground mass production in the non-grafted and self-grafted pepper plants. The least biomass loss was found in the pepper plants of the Adige variety, grafted onto the A25 rootstock, which have been previously defined as tolerant, while Adige has been described as drought-sensitive (Penella et al., 2017). Different drought stress effects were observed for the growth parameters in both roots and leaves; the growth inhibition of roots (DW) was lower (a reduction of 19% in A, 11% in A/A and 13% in A/A25 compared to their controls) *versus* leaves at a low osmotic potential of the solution (a reduction of 40% in A, 31% in A/A and a non-effect in A/A25). This result indicated that leaf growth was more sensitive to drought stress compared to roots, except for A/A25 where the effect of PEG was not detected in leaves. This differential response in the growth inhibition between roots and leaves under drought stress (in our results for plants A and A/A) has been observed by several authors (Westgate and Boyer, 1985; Sharp et al., 1994; Hsiao and Xu, 2000). It resulted in a sudden reduction of Ψw in roots, which allowed water to enter. Then solutes had to enter to prevent dilution and maintain the osmotic forces needed for growth. On the contrary in leaves, osmotic adjustment occurs by slowly limiting their expansion (Hsiao and Xu, 2000). Beyond the biophysical aspect, there is compelling evidence that ABA accumulation under drought stress plays a pivotal role by inhibiting shoot growth, as in plants A and A/A, while a minor effect is observed in roots (Sharp et al., 1994). Nevertheless, the behavior of plants A/A25 did not match these results. Currently, we have no plausible explanation for this. All these results suggest that scion growth is largely dependent on rootstock tolerance to drought stress.

The growth reductions mediated by drought stress evidence a series of changes in various biochemical processes, such as photosynthesis (Urban et al., 2017). Net CO_2_ assimilation decreased suddenly 24 h after adding PEG to A, followed by plants A/A, while A/A25 maintained similar *A*_N_ values to the control up to 48 h (2 DAT); afterward *A*_N_ significantly lowered until the end of the experiment, but values were higher in A/A25 than in A and A/A under PEG, with sustained photosynthetic activity during drought (Rouphael et al., 2008; Penella et al., 2014b). These results agree with previous findings in which grafting onto a tolerant rootstock improved the photosynthesis performance of plants under drought stress (Schwarz et al., 2010; Penella et al., 2014b). In A/A25, the decrease in *A*_N_ on 4 DAT and 7 DAT was accompanied by a significant decrease in stomatal conductance, although the decline in g_s_ occurred earlier than the reduction in CO_2_ fixation (1 DAT and 2 DAT). We could assume that stomata closure probably did not limit CO_2_ acquisition by leaves under drought stress within the first two time frames (Delfine et al., 2002). The decrease and the subsequent maintenance of the intercellular CO_2_ concentration (*C*_i_) in A/A25 under drought stress (compared to the A/A25 control plants) implied that stomatal limitations were responsible mainly for the reduction in *A*_N_ by drought stress (Delfine et al., 2002; Rouphael et al., 2008). In plants A and A/A under drought stress, the drastic decline of *A*_N_ was in line with the strong stomata closure from the beginning to the end when time measurements were taken (more marked in the A plants). However, *C*_i_ suddenly lowered on 1 DAT, 2 DAT, and 4 DAT, with a significant increase at the end of the experiment compared to the controls, which implies the existence of stomata and non-stomatal limitations related with changes in the cellular carbon metabolism, which can affect the growth mediated by reducing the biochemical capacity for carbon assimilation and utilization (Flexas et al., 2004; Reddy et al., 2004; He et al., 2009). All this was true, except for plants A/A25, which showed no significant differences between plants under the control and PEG conditions (7 DAT).

Apart from the discussed changes in carbon assimilation, drought stress may affect several nitrogen metabolism stages (Feller and Vaseva, 2014; Penella et al., 2014b) by inducing visible effects on biomass. One important step is the assimilation of nitrate into organic compounds. The activity of the first enzyme involved in this process, NR, was negatively influenced by drought; nitrogen assimilation is also coordinated with carbon assimilation as photosynthesis is required for NR activation (Kaiser and Huber, 2001). Nitrate reduction is sensitive to stomatal resistance and *g*_s_ decreases in plants under drought stress to preserve water loss when not only *A*_N_ drops, but NR also becomes less active (Kaiser and Huber, 2001; Yousfi et al., 2012; Penella et al., 2014b). In relation to this coordination between NR activity and *A*_N_, we observed a quick decrease in NR of the A plant leaves 24 h after inducing drought stress, and after 48 h in the A/A plants, while the A/A25 plants preserved their activity until 4 DAT. At the end of the experiment, the A plants, followed by A/A, did not show any NR activity and *g*_s_ dropped, while A/A25 maintained 64% enzyme activity and sustained *g*_s_ compared to the control. Some non-stomatal effects, such as reduced nitrate availability in plants, could inhibit NR gene transcription and decrease the stability of NR-mRNAs or post-translational factors, including inactivation through protein phosphorylation, and the induction of proteases could rapidly occur by decreasing NR activity as a result of drought stress (Ferrario et al., 1995; Lillo et al., 2004; Correia et al., 2005). In most herbaceous plants, NO_3_^-^ assimilation takes place predominantly in leaves (Scheurwater et al., 2002). Accordingly, NR activity was greater in leaves than roots in all the plant combinations and treatments in our study. Nevertheless, the greatest root-NR activity was observed under drought stress, and maximum activities were displayed for the A plants, followed by the A/A plants, where lower leaf NR seemed to be partly compensated by an increase in root NR (Lexa and Cheeseman, 1997; Penella et al., 2014b). Drought stress can decrease nitrate uptake by roots. Besides, the transfer of NO_3_^-^ to leaves can be limited by stomata closure. Thus transpiration (data not shown) diminished as part of nitrate can reduce in roots. This happened in plants A and A/A as A/A25 had a higher root NR on 1 DAT and 2 DAT, be it to a lesser extent, because the higher g_s_ allowed NO_3_^-^ transport to leaves for its reduction. This role of NR in leaves and roots under drought stress has been observed in grafted pepper (Penella et al., 2014b), pea (Lexa and Cheeseman, 1997) or wheat plants (Yousfi et al., 2012).

The decrease in Ψs during the PEG treatment could be a consequence of less water content in tissue and/or through the active osmotic adjustment involving the net accumulation of a range of osmotically active molecules in response to a drop in the Ψw in their environment. In pepper, short-term exposure with different osmotic potentials of the nutrient solution (Navarro et al., 2003; Martínez-Ballesta et al., 2004; Silva et al., 2008) showed that the decrease in Ψw was not compensated by a reduction in Ψs and, as a result, the osmotic adjustment was negligible. According to our results, the decrease in the leaves of Ψw, Ψs and stomatal conductance in the PEG treatment showed that water uptake could not balance water loss and decreased RWC, along with there being fewer biochemical functions in A and A/A. This could indicate that osmotic adjustment was insignificant. Nevertheless, RWC was maintained in A/A25 with PEG addition, which could indicate an osmotic adjustment at the end of the experiment, when Ψw remained constant and Ψs decreased. Similar results have been obtained by Penella et al. (2014b). A deep root system and higher root biomass have shown as beneficial effects for acquiring water (Koevoets et al., 2016), and could be one of the reasons for the unchanged RWC values in the A/A25 plants noted throughout the experiment. They suggest a typical conservative water strategy (Tardieu and Simonneau, 1998; García-Sánchez et al., 2010; Sade et al., 2012; Penella et al., 2016). This would lead to more drought tolerance and would allow photosynthesis preservation, improved absorption, upward transfer and NO_3_^-^ accumulation in leaves. Similar results have been shown in the susceptible tomato scion “Josefina” grafted onto drought stress-tolerant “Zarina” rootstocks (Sánchez-Rodríguez et al., 2013). However, the acclimation rate and drought stress duration are key factors that can influence depending on plant varieties and/or age.

This limited water uptake and CO_2_ uptake in plants A and A/A under PEG conditions accelerated oxidative damage by producing ROS (Asada, 1999). Hydrogen peroxide is one type of ROS produced as a result of the dismutation of the superoxide radical, and a higher concentration damages both the cell and the whole plant to result in lipid peroxidation and membrane injury (Sairam and Srivastava, 2001). In the non-grafted tomato plants (Rivero et al., 2003b), a major increase in H_2_O_2_ was observed under thermal shock stress compared with the grafted plants. In A, and to a lesser extent in the A/A pepper plants, drought stress caused excessive H_2_O_2_ accumulation in leaves, as well as high lipid peroxidation compared with the A/A25 plants and their controls. This result suggests that H_2_O_2_ in leaves is largely dependent on the adaptability of roots to drought stress. This effect has been observed in cucumber plants grafted onto fig leaf gourd (*Cucumis ficifolia*) or onto luffa (*Luffa cylindrical*), where a significantly low H_2_O_2_ concentration alleviated membrane lipid peroxidation at high temperature (Li et al., 2014) compared with non-grafted plants. The authors attributed this reduction to increased CO_2_ assimilation and ROS-scavenging activity. In roots, the maximum H_2_O_2_ concentration was observed on 2 DAT for the A-PEG and A/A25-control plants, but its concentration was 10-fold lower than in leaves. Afterward, levels lowered in this organ, whose concentration in leaves was “amplified.” As a result of increasing H_2_O_2_, the MDA concentration was seen to be enhanced in both roots and leaves, with less lipid peroxidation damage in roots. A lower H_2_O_2_ concentration in A/A25 could be due to less ROS production or to the more efficient detoxification of this compound (Rivero et al., 2003b). Antioxidant activity was evaluated by the effect of the extracted samples on the DPPH radical. Under the PEG conditions and at the end of the experiment, plants A/A25 showed the least radical scavenging activity, which indicates that the alleviation of oxidative stress occurred to a lesser extent in the A/A25 plants compared with the greatest activity in the A plants, followed by the A/A plants. In response to drought stress, plants can accumulate a wide range of antioxidants, including phenolic compounds (Keleş and Öncel, 2002). Phenolic compounds exhibit antioxidant activity by inactivating lipid free radicals or preventing the decomposition of hydroperoxides into free radicals (Pokorny, 2001). Our results showed that with PEG addition, the synthesis of phenols increased, but the most marked rise took place in the A plants, followed by A/A and A/A25. Even though the stimulation of antioxidant activity in plants A and A/A occurred simultaneously with higher phenol concentrations under drought conditions, an imbalance between ROS generation and scavenging systems might have occurred as the highest H_2_O_2_ and MDA levels was confirmed.

According to these results, grafting itself (A/A plants) has a slightly positive effect on the physiological parameters measured under drought stress compared with the A plants, probably due to enhanced endogenous hormone production as a result of the grafting *per se* incision, which may influence the transport of hormones between roots and the scion that, in turn, could alter weak responses (Aloni et al., 2008; Savvas et al., 2011; Orsini et al., 2013).

## Conclusion

To conclude, our results suggest that plants A/A25 were more tolerant to drought stress given the response made in several physiological processes in the short term, which were maintained for 7 days under water stress, and even beyond this time given better fruit production (Penella et al., 2017). Growth preservation in plants A/A25 after PEG addition was associated with maintained CO_2_ assimilation and partly open stomata, which allowed water uptake and preserved RWC. The conservative water strategy involved minor oxidative stress as demonstrated by the lower H_2_O_2_ concentration and diminished membrane lipid peroxidation. These results could be attributed to the capacity to maintain shoot growth by the root system’s conservative tolerance traits under drought stress. Consequently, the grafting of commercial cultivars onto drought-tolerant rootstock(s) such as A/A25 can be considered a valid strategy to improve drought stress tolerance. Nevertheless, other mechanisms, like the hormone signaling cascade (Cantero-Navarro et al., 2016) or the mobility of genetic components (Haroldsen et al., 2012), which were not contemplated herein, could also explain the improved drought tolerance of these grafted plants, and should be studied in future works.

## Author Contributions

LL-S, CP, and ÁC conceived and designed the experiments. LL-S, GC-S, GVS, CP, and ÁC performed the experiments. AS, SL-G, LL-S, and ÁC analyzed the data and discussed the results of this study. ÁC wrote the paper. All the authors read and approved the manuscript.

## Conflict of Interest Statement

The authors declare that the research was conducted in the absence of any commercial or financial relationships that could be construed as a potential conflict of interest.
